# Development of a single-cell atlas for woodland strawberry (*Fragaria vesca*) leaves during early *Botrytis cinerea* infection using single-cell RNA-seq

**DOI:** 10.1093/hr/uhab055

**Published:** 2022-01-19

**Authors:** Yibo Bai, Hui Liu, Haimeng Lyu, Liyao Su, Jinsong Xiong, Zong-Ming (Max) Cheng

**Affiliations:** College of Horticulture, Nanjing Agricultural University, Nanjing 210095, China

## Abstract

Pathogen invasion leads to fast, local-to-systemic signal transduction that initiates plant defense responses. Despite tremendous progress in past decades, aspects of this process remain unknown, such as which cell types respond first and how signals are transferred among cell types. Here, we used single-cell RNA-seq of >50 000 single cells to document the gene expression landscape in leaves of woodland strawberry during infection by *Botrytis cinerea* and identify major cell types. We constructed a single-cell atlas and characterized the distinct gene expression patterns of hydathode, epidermal, and mesophyll cells during the incubation period of *B. cinerea* infection. Pseudotime trajectory analysis revealed signals of the transition from normal functioning to defense response in epidermal and mesophyll cells upon *B. cinerea* infection. Genes related to disease resistance showed different expression patterns among cell types: disease resistance-related genes and genes encoding transcription factors were highly expressed in individual cell types and interacted to trigger plant systemic immunity to *B. cinerea*. This is the first report to document the single-cell transcriptional landscape of the plant pathogenic invasion process; it provides new insights into the holistic dynamics of host–pathogen interactions and can guide the identification of genes and the formulation of strategies for resistant cultivar development.

## Introduction

Plant pathogens cause billions of dollars in economic losses by reducing or eliminating crop yield or quality. Plants respond to external infections and defend themselves against pathogen invasion by initiating two branches of the innate immune system [1]. The first branch identifies and responds to microbial molecules, including those from non-pathogens, by means of pattern recognition receptors located on the surface of cell membranes. Pattern recognition receptors recognize pathogen-associated molecular patterns (PMAPs) and induce PAMP-triggered immunity (PTI) in plants [2]. The second branch of the innate immune system responds to pathogen factors. When pathogenic bacteria escape the PTI response through secreted protein factors, plant receptor R proteins recognize the secreted effector proteins and induce effector-triggered immunity by interacting with them directly or indirectly [1].

This process has been studied in *Botrytis cinerea*, a necrotrophic fungus of global importance that causes grey mold disease and infects >1000 species worldwide, including >200 crop species [3, 4], such as grapes and strawberry. In hot, humid environments, conidia of *B. cinerea* invade through stomata, hydathodes, or wounds [5], and early disease diagnosis may protect plants from damage [6]. In recent years, mechanisms of strawberry defense against the early stage of gray mold disease have been studied by various omics approaches [7–9], and conventional omics have been used to characterize the response to *B. cinerea* at the whole-organ level. However, such experiments can obscure the characteristics of different cell populations, and cell heterogeneity in the biotic stress response may affect disease assessment and targeted treatment [10]. For this reason, single-cell sequencing technology has potential utility for studying the heterogeneity of cell responses during the pathogen incubation period of plants.

Single-cell technology has recently been applied to plant biology because of its ability to map the transcriptional landscape with a high degree of spatial resolution. With this technology, it is possible to explore the heterogeneity among different tissue and cell types and to identify unknown cell types. This enables predictions of the developmental trajectory based on different states of the cells [11, 12]. The limited research to date has established protocols for distinguishing among various heterogeneous cell types, and these protocols have been applied to *Arabidopsis* root system development, stomatal development in *Arabidopsis* leaves, and construction of a single-cell atlas for maize ears [13–15]. Single-cell sequencing technology has also been applied to studies of plant stress. For example, the root cells of *Arabidopsis* exhibit heterogeneous responses to heat stress [16]. Under low-phosphate conditions, *Arabidopsis* roots increase the density of vascular cells and root hairs to maintain normal physiological activities [17]. Under high salinity, low nitrogen, and iron deficiency, the roots and above-ground parts of rice seedlings show heterogeneity in stress response among cell types [18]. However, there has been no single-cell research on plants under disease stress. In this study, we used single-cell RNA-seq (scRNA-seq) to construct a single-cell transcriptome atlas of woodland strawberry leaves at three stages of *B. cinerea* infection: 0 h post-inoculation (hpi, mock), 6 hpi (S6), and 12 hpi (S12). We identified the features of cells and molecules in woodland strawberry leaves at different disease stages and characterized cells from primary infection sites, such as hydathode, upper epidermal, and mesophyll cells, which respond first to *B. cinerea* infection. In addition, we analyzed the pseudotime trajectories of upper epidermal and mesophyll cells across different lesions. Finally, we documented the expression profiles of disease-related genes and genes that encode transcription factors (TFs). Our atlas provides new insights into cellular heterogeneity during plant–pathogen interactions.

## Results

### A single-cell atlas of woodland strawberry leaves

**Figure 1:**
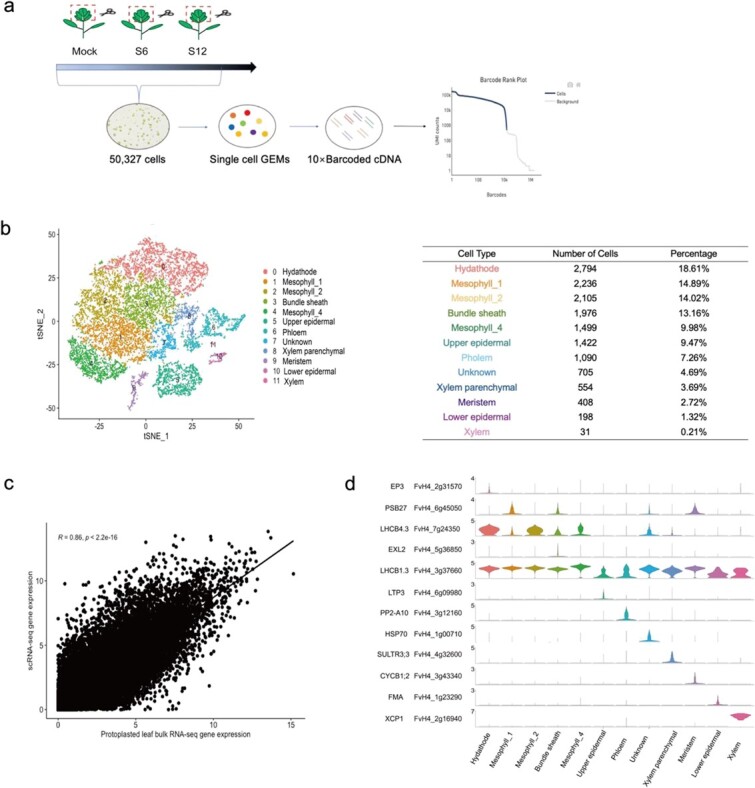
Cellular heterogeneity cluster analysis of the *F. vesca* leaf single-cell transcriptome. **a** Details of single-cell library preparation. Protoplasts were obtained by enzymatic hydrolysis. mRNA released by rupture of a single-cell suspension was combined with gel bead and emulsion to form GEMs. The mRNA of the cell was independently reverse-transcribed in each GEM, and tagged cDNA will be mixed and amplified to library construction. Mock, 0 hpi; S6, 6 hpi; S12, 12 hpi. **b** t-SNE visualization divides 15 018 *F. vesca* leaf cells into 12 clusters. **c** Correlation analysis of scRNA-seq and bulk RNA-seq gene expression. **d** Violin plots of representative cluster-specific marker genes in different cell types.

To characterize the single-cell profiles of woodland strawberry, we performed scRNA-seq on the first true leaves of ‘Hawaii 4’ seedlings (Fig. 1a). Large-scale single-cell isolation yielded at least 20 000 cells, which that were combined with gel beads carrying cell tag sequences and wrapped in droplets (Fig. 1a). After removing double cells and low-quality cells, we obtained a single-cell transcriptome of 15 039 cells from the mock (0 hpi) sample; it contained 861 223 068 reads, 65.50% of which mapped to the *Fragaria vesca* v4.01 genome. The median number of unique molecular identifiers (UMIs) per cell was 15 233, and an average of 3344 genes were expressed per cell. Expression of 21 392 genes was detected in the mock sample (Supplementary Table S1). When the bulk RNA-seq and scRNA-seq data were compared, gene expression in the bulk-sequenced and single-cell-sequenced true leaves showed a significant correlation (*R* = 0.86, *P* < 2.2e^−16^). In general, the scRNA-seq data obtained in this experiment were of high quality (Fig. 1c, Supplementary Fig. S2a and b).

After filtering and removal of mitochondria and chloroplast organelles, 15 018 cells were used for dimensionality reduction and divided into 12 cell clusters based on their heterogeneity using the *t*-distributed stochastic neighbor embedded (t-SNE) tool (Fig. 1b). Because there are no previous reports on cell heterogeneity in strawberry, the *Arabidopsis* orthologs of cluster-specific genes were used to annotate these clusters (Supplementary Table S3) [19–22]. EP3 and WRKY29 are highly expressed and specific to hydathode cells [22]. In epidermal cells, we identified lipid-transfer proteins (LTP1, LTP3) and 3-ketoacyl-CoA synthase 6 (CER6) as marker genes [23–26]. The cell cluster enriched in waxy layer-related genes was identified as the upper epidermal cluster, and the cluster enriched in FMA (basic helix-loop-helix transcription factor) was identified as the lower epidermal cluster. Ribulose bisphosphate carboxylase small chain 1A (RBCS1A), chlorophyll a–b binding protein of the LHCII type (CAB1), chlorophyll a–b binding protein (LHCA2), and light harvesting complex photosystem II subunit 6 (LHCB6) are known marker genes for mesophyll cells [14, 27–29]. Calmodulin-like protein 1 and phloem protein 2-A10 (PP2-A10) served as markers for phloem cells [20, 25]. Cyclin-dependent kinase B2;1 (CDKB2;1), CYCLIN B2;4 (CYCB2;4), and G2/mitotic-specific cyclin (CYCB1;2) are highly expressed in meristems and were used as meristem cell marker genes [30]. Chitinase-like protein 2 (CTL2) and xylem cysteine peptidase 1 (XCP1) were used as representative xylem marker genes [21, 25]. Bundle sheath cells were enriched in EXORDIUM like 2 (EXL2) and NAD(P)-binding Rossmann fold superfamily protein (VEP1) [31]. MYB domain protein 59 and sulfate transporter 91 (SULTR3;3) were used as marker genes for xylem parenchymal cells [31]. Based on these markers, we identified 12 cell types: hydathode cells (cluster 0), mesophyll_1 (cluster 1), mesophyll_2 (cluster 2), mesophyll_4 (cluster 4), bundle sheath cells (cluster 3), upper epidermal cells (cluster 5), phloem cells (cluster 6), lower epidermal cells (cluster 10), xylem parenchymal cells (cluster 8), meristem cells (cluster 9), and xylem cells (cluster 11). Heat shock protein was enriched in cluster 7, but it was not possible to determine the cell type of this cluster using the current marker genes (Fig. 1d).

Gene Ontogeny (GO) enrichment was used to further classify each cell type (Supplementary Fig. S3c); different cell types were enriched in distinct sets of biological processes (*P*_adj_ ≤ 0.05). For example, mesophyll cells were rich in biological processes related to photosynthesis, and epidermal cells were mainly enriched in biological processes related to fatty acid synthesis [24]. As a conductive tissue, the vasculature was enriched in genes that participate mainly in the conduction of various plant signal molecules and in the transport of metal ions [32].

### Identification of different cell types

**Figure 2:**
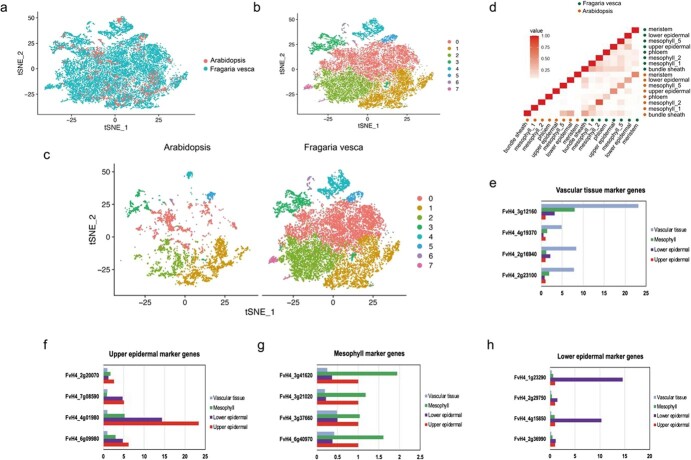
Comparison of woodland strawberry and *Arabidopsis* leaves and overview of tissue-specific gene expression in woodland strawberry. **a, b** t-SNE visualization of *F. vesca* and *Arabidopsis* cell clusters after integration by species (**a**) or cell type (**b**). **c** t-SNE plot of separated of *F. vesca* and *Arabidopsis* single cells. **d** Pearson’s correlation coefficients of gene expression in woodland strawberry (dark green) and *Arabidopsis* (orange) cell types. **e**–**h** Expression of known tissue-specific marker genes for vascular (**e**), upper epidermal (**f**), mesophyll (**g**), and lower epidermal tissue (**h**).

To assess the conservation of cell type gene expression between woodland strawberry and *Arabidopsis*, we compared the woodland strawberry scRNA-seq data with the published *Arabidopsis* leaf dataset [31] to identify single-copy orthologs using Orthofinder (Supplementary Table S4). We integrated the single-cell data from *Arabidopsis* and woodland strawberry, then grouped the cells into eight clusters through dimensionality reduction (Fig. 2a and b). t-SNE visualization and Pearson’s correlation coefficients showed that there was high homology between cell types of *Arabidopsis* and woodland strawberry (Fig. 2c and d). Furthermore, shared marker genes between the two species further confirmed the conservation of homologous cell types (Supplementary Fig. S4a). For instance, glycosylphosphatidylinositol-anchor lipid transfer protein 1 (LTPG1) was shown to be highly expressed in the epidermal cells and to participate in wax monomer transport [26].

To confirm the expression of different tissue-specific genes, woodland strawberry leaves were divided into upper epidermal, lower epidermal, mesophyll, and vascular tissues by laser microdissection (Fig. 2e–h). Using quantitative real-time PCR (RT–qPCR), we found that *FvH4_4g01980* (KCS2) and *FvH4_6g09980* (LTP3) were highly expressed in the upper epidermal tissue, and *FvH4_1g23290* and *FvH4_4g15850*, which promoted differentiation of stomatal guard cells, were specifically expressed in the lower epidermal tissue. *FvH4_3g41620* (PSAG) and *FvH4_6g40970* (LHCB2.1) were highly expressed in mesophyll cells, and *FvH4_3g12160* (PP2-A10) and *FvH4_2g16940* (XCP1) were highly expressed in vascular cells (Fig. 2e–h). These results further verify the accuracy of the strawberry leaf cell-type classification.

### Single-cell analysis of *F. vesca* responses to *B. cinerea* infection

**Figure 3:**
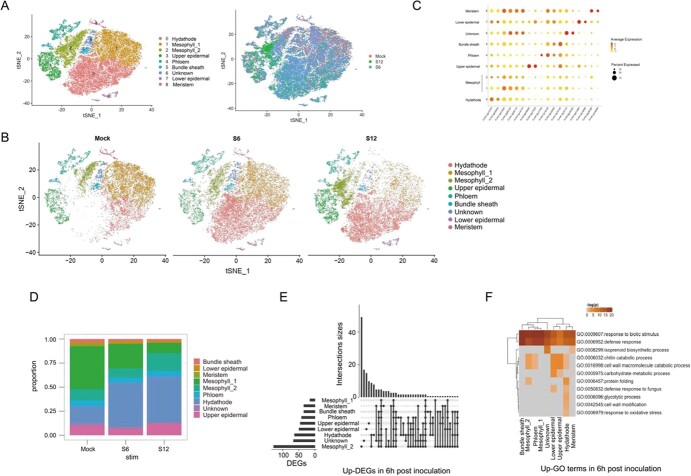
Single-cell transcriptome analysis of infected and control samples. **a** t-SNE plot of 50 327 woodland strawberry leaf cells from different infection stages (mock, 15 039 cells; S6, 18 223 cells; S12, 17 065 cells) and cell types (right). S6 represents the sample taken at 6 h after inoculation and S12 represents the sample taken at 12 h after inoculation with *B. cinerea*. **b** t-SNE plot of separated mock, S6, and S12 single cells. **c** Expression patterns of cell-type marker genes for each cell cluster. Dot diameter indicates the percentage of cluster cells that expressed the gene. Color indicates the average expression across cells in that cluster. **d** Bar plot showing the relative proportion of each cell type in mock, S6, and S12 samples. **e** Upset plot of upregulated DEGs at 6 hpi by *B. cinerea*. The bar plot on the left shows the number of upregulated DEGs for each cell type. The upper bar plots show the number of upregulated DEGs. (∣logFC∣ ≥ 0.8, FDR ≤ .01). **f** GO terms enriched in upregulated DEGs at 6 hpi in multiple cell types (FDR ≤ .05).

A major challenge in studying the plant responses to biotic stress is the inconsistent degree of reactions among cell types. To determine how each strawberry leaf cell type responds to *B. cinerea* and how the systemic signals are generated and propagated from the infection site [6], we explored differences in leaf cell responses to the pathogen. At 6 hpi (S6), when the hyphae of *B. cinerea* had gathered on the surface of the leaves (Supplementary Fig. S1e), we captured 18 223 cells with a median expression of 3144 genes per cell and a total of expressed 21 629 genes; 64.5% of the sequenced reads could be mapped to the *F. vesca* v4.01 genome (Supplementary Table S1). At 12 hpi (S12), when the *B. cinerea* hyphae had penetrated the leaf epidermis (Supplementary Fig. S1f), we captured 17 065 cells with a median expression of 3199 genes per cell and a total of 21 555 expressed genes, 65.4% of the sequenced reads could be mapped to the genome (Supplementary Table S1). To study cellular heterogeneity during pathogen infection, we integrated the three samples and corrected batch effects using CCA+ (Fig. 3a and b). Nine cell types were identified based on cluster-specific marker genes (Fig. 3c). The proportions of these cell types changed when leaves were infected with *B. cinerea*. For instance, the proportion of hydathode cells in the transcriptome was 16.6 and 16.3% at 6 and 12 hpi, an increase of 5% compared with the mock sample (Fig. 3d). As the treatment duration increased, the transcriptome proportion of mesophyll_1 cells decreased from 13 to 9% at 6 hpi and 3.5% at 12 hpi. These results were suggestive of cellular and physiological differences at different periods of *B. cinerea* infection.

To provide insights into the gene expression pattern in each cell type during the *B. cinerea* infection process, we identified differentially expressed genes (DEGs) between infection time points for specific cell types and performed GO analysis of the DEG sets (Fig. 3e and f, Supplementary Fig. S5). The number of DEGs in each cell type increased over the course of the *B. cinerea* infection process. The GO terms ‘response to biotic stimulus’ and ‘defense response’ were enriched in DEGs of all cell types at 6 and 12 hpi relative to 0 hpi. Some biological process GO terms were enriched in DEGs from specific cell types, e.g. ‘defense response to fungus’ was enriched in DEGs from epidermal and hydathode cells at 6 hpi. ‘Response to oxidative stress’ was enriched in DEGs from hydathode cells at 6 hpi, and this enrichment intensified as time elapsed (Supplementary Fig. S5). At 12 hpi, ‘response to oxidative stress’ was enriched in DEGs from multiple cell types: meristem, hydathode, mesophyll_2, and bundle sheath. Furthermore, the GO terms ‘response to stress’ and ‘sodium ion transport’ were also enriched in specific cell types at 12 hpi.

### Characterization of single-cell expression profiles for cell lineages first infected by *B. cinerea*

**Figure 4:**
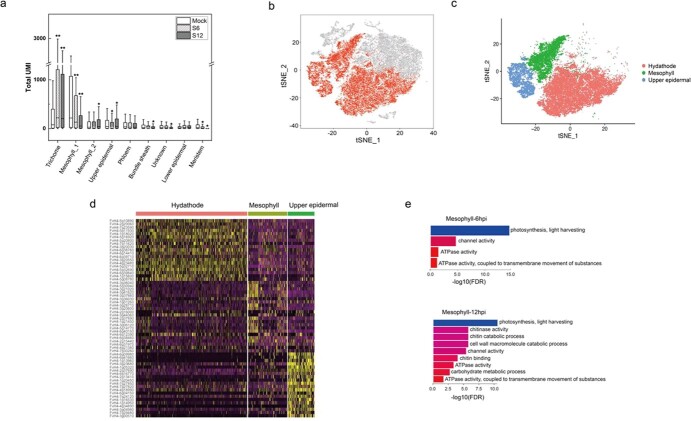
scRNA profiles for cell types that respond first to the infection stimulus across different infection stages. **a** Distribution of total UMI for individual cell types in integrated single-cell data. **b, c** t-SNE plots showing the distribution of hydathode, upper epidermal, and mesophyll cells for integrated samples. **d** Heatmap showing the expression of selected functionally relevant genes that were differentially expressed between the three cluster types. **e** The most enriched GO terms in upregulated genes of mesophyll cells in 6 and 12 hpi samples (FDR ≤ .05).

To identify the cell types that were first infected by *B. cinerea*, we analyzed the percentage of cells and UMI values across multiple lesions. In general, we observed that the proportions and UMI values of hydathode, upper epidermal, and mesophyll cells increased significantly during lesion progression and the hydathode, mesophyll_2, and upper epidermal cell types appeared to respond first to the *B. cinerea* stimulus (Figs 3d and 4a). Surprisingly, the hydathode, mesophyll and upper epidermal cells showed distinct expression patterns during the *B. cinerea* infection process (Fig. 4c); genes involved in transferase activity and polysaccharide catabolic process were expressed mainly in hydathode cells, whereas genes involved in signaling pathways of translation and binding were expressed mainly in the mesophyll_2 cells, and genes involved in channel activity and response to stress signaling pathways were expressed mainly in the upper epidermal cells (Supplementary Table S7). Expression of genes in the pathways related to disease symptom development increased in the upper epidermal and mesophyll cells as the infection progressed (Fig. 4, Supplementary Fig. S6).

### Cell trajectory analysis of epidermal and mesophyll cells during the different stages of *B. cinerea* infection

**Figure 5:**
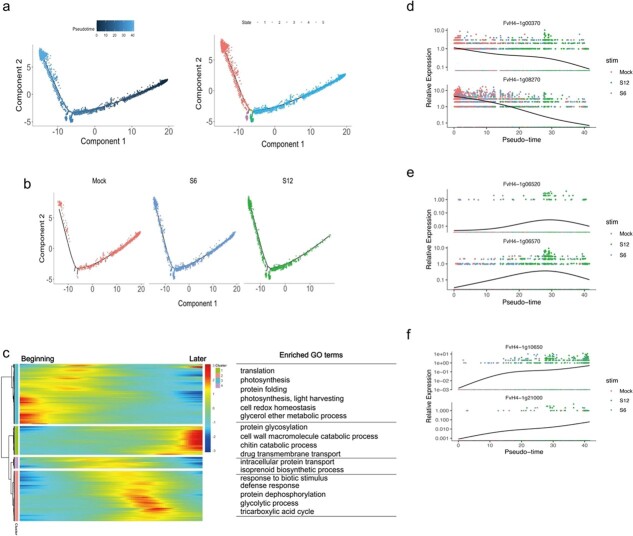
Pseudotime analysis of mesophyll cells in mock (0 hpi), S6 (6 hpi) and S12 (12 hpi) samples. **a** Pseudotime trajectory of mesophyll cells. Each dot represents a single cell. Color represents the pseudotime score (left). Color represents different states (right). **b** Mesophyll cell distribution on the trajectory for mock, S6 and S12 samples. **c** Pseudotime heatmap of GO analysis of differentially expressed genes (FDR ≤ .05). Color bar indicates the relative gene expression level. **d**–**f** Gene expression kinetics of representative genes along a pseudotime progression in the beginning (**d**), middle (**e**) and later (**f**) stage.

To evaluate the differences in expression profiles at different stages, pseudotime analysis was performed on mesophyll and epidermal cells. For mesophyll cells, the three samples were projected to three ends of the pseudotime trajectory, including five trajectory states, and gathered mainly at one of the large ends (Fig. 5a). We separated the cell trajectories of the three stages and found that the cells shifted gradually from state 4 to state 1 as processing time increased (Fig. 5b), which summarized the developmental process of mesophyll cells stimulated by *B. cinerea.* The gene expression patterns of cells in different states were calculated in pseudotime order using Monocle 2. Differentially expressed genes were divided into four clusters, reflecting changes in differential gene expression from the beginning to the end of the pseudotime. GO terms related to photosynthesis and translation which function in mesophyll cells were enriched at the beginning of the pseudotime. Genes related to biotic stress response, such as *FvH4_1g06520* and *FvH4_1g06570*, were enriched in the middle stage of pseudotime (Fig. 5e), whereas genes related to protein glycosylation and chitin catabolic processes, such as *FvH4_1g10650* and *FvH4_1g21000*, were particularly prominent at the later stage (Fig. 5f).

Similarly, the upper epidermal cells from different time points had two distinct trajectories (Supplementary Fig. S7a and b). The DEGs across branch points were divided into three clusters (Supplementary Fig. S7c and d), Cluster 3-1 contained mainly genes involved in translation and fatty acid biosynthetic processes, such as *FvH4_1g18550* and *FvH4_4g06700*, which were indicative of the function of epidermal cells (Supplementary Fig. S7e). Cluster 1 and cluster 3-2 were enriched in defense response and metabolic genes, consistent with the transition in epidermal cell state during the infection response (Supplementary Fig. S7c and d).

### Changes in expression of defense-related genes during lesion development

**Figure 6:**
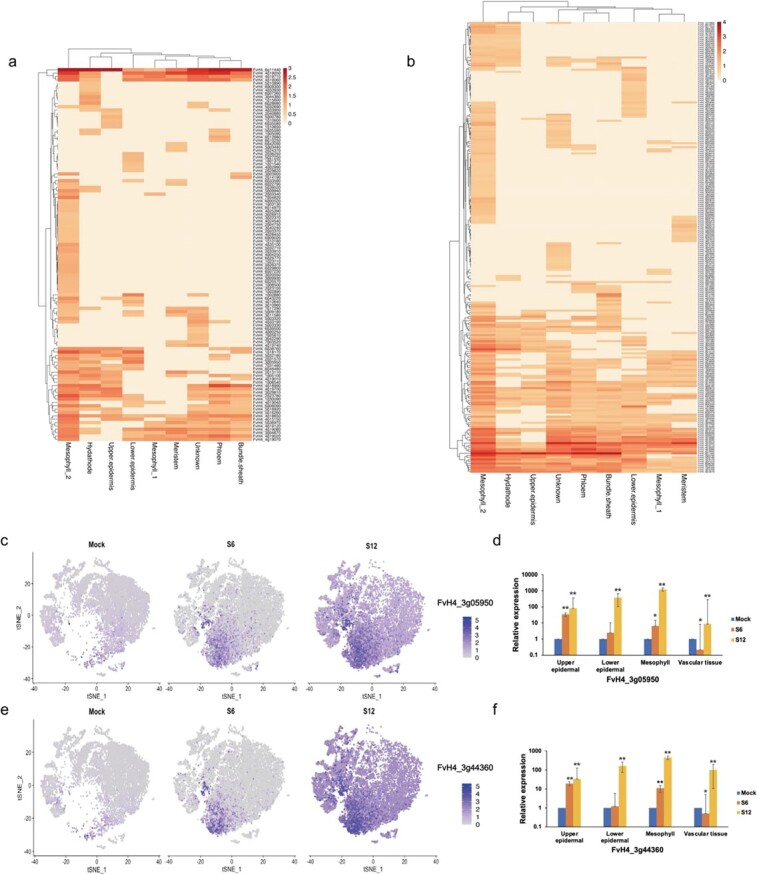
Resistance-related gene expression at 0 (mock), 6 (S6), and 12 hpi (S12) with *B. cinerea*. **a** Heatmap showing the expression of resistance-related genes in each cell type at 6 hpi. **b** Heatmap showing the expression of resistance-related genes in each cell type at 12 hpi. **c**, **d** FeaturePlot (**c**) and RT–qPCR (**d**) showing the expression distribution of *FvH4_3g05950* (PR4) in different processing stages (Student’s *t-*test, ^*^P < .05 and ^**^P < .01). **e**, **f** FeaturePlot (**e**) and RT–qPCR (**f**) showing the expression distribution of *FvH4_3g44360* (PA2) in different processing stages (Student’s *t-*test, ^*^P < .05 and ^**^P < .01).

We observed significant upregulation of genes encoding receptor-like proteins (*FvH4_1g01370*) in the lower epidermal cells during the incubation period of *B. cinerea* infection (Fig. 6a and b). Leucine-rich repeat (LRR) family proteins were expressed in mesophyll_2 cells. Subsequently, we identified other genes related to recognition and signaling. For example, *FvH4_6g09300*, related to calmodulin, was expressed mainly in hydathode cells at 6 hpi. However, at 12 hpi, *FvH4_6g09300* (CML42) was expressed in almost cell types, demonstrating that genes expressed in response to *B. cinerea* may be upregulated in one or several cell types during the incubation period of the infection (Supplementary Fig. S8a).

To further investigate the expression patterns of defense-related genes, we measured their expression levels in different cell types. The disease-related protein PR4 (*FvH4_3g05950*) was expressed in hydathode, mesophyll_2, and upper epidermal cells at 6 hpi, and its expression in other cell types increased as the infection progressed to 12 hpi (Fig. 6c and d). Peroxidase PA2 (*FvH4_3g44360*) was first expressed in hydathode cells at 6 hpi (Fig. 6e and f). With increasing infection duration, its expression level gradually increased in other cell types. These results were consistent with those shown in Fig. 4a, implying that hydathode, upper epidermal, and mesophyll cells have the highest levels of disease response-related transcriptional variation during early infection.

### Transcriptional regulatory network during the incubation period of strawberry infection by *B. cinerea*

**Figure 7:**
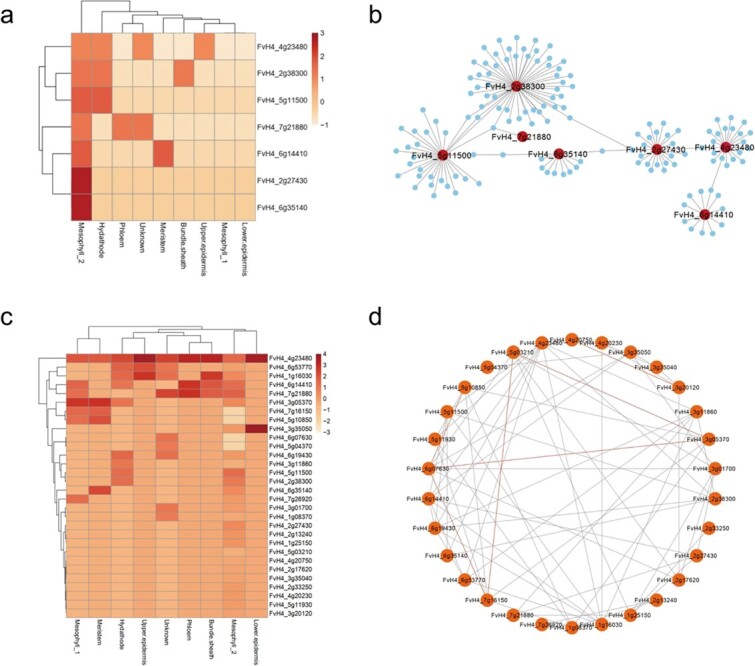
Analysis of core TFs in different stages of infection. **a** Heatmap showing the expression of TFs in each cell type at 6 hpi. **b** Protein–protein interaction network of upregulated TFs expressed in response to *B. cinerea* at 6 hpi. **c** Heatmap showing the expression of TFs in each cell type at 12 hpi. **d** Co-expression network of TFs upregulated expressed in response to *B. cinerea* at 12 hpi.

TFs were also expressed in different cell types during the infection process. At 6 hpi, members of the NAC, HSP, WRKY, VQ, and TIFY TF families were highly expressed in woodland strawberry (Fig. 7a). Interestingly, HSP90 (*FvH4_2g38300*) was expressed mainly in hydathode and bundle sheath cells, WRKY75 (*FvH4_4g23480*) was expressed mainly in the upper epidermal cells, and ZAT11 (*FvH4_6g14410*) was highly expressed in the phloem. Protein–protein interaction network analysis demonstrated the interaction of TFs expressed in different cell types at 6 hpi; these TFs presumably work together to regulate the strawberry immune response to defend against *B. cinerea* (Fig. 7b)*.* Subsequently, we examined a clustered heatmap of TF expression at 12 hpi and found that the WRKY family gene, WRKY 75 (*FvH4_4g23480*) exhibited high expression in each cell type (Fig. 7c). Likewise, *FvH4_6g53770* (WRKY DNA-binding protein), *FvH4_1g16030* (HSF4), *FvH4_6g14410* (ZAT11), and *FvH4_7g21880* (ZAT12) had high levels of expression in most cell types. *FvH4_3g11860* (NAC042) and *FvH4_3g35050* (zinc finger protein) were specifically expressed in the hydathode and lower epidermal cells, respectively. To identify the regulatory relationships among upregulated TFs, we constructed a co-expression network of upregulated TFs expressed in response to *B. cinerea* at 12 hpi (Fig. 7d). The 30 TFs were connected to each other through 69 edges, and TFs that were highly expressed in multiple cell types, such as *FvH4_4g23480* (WRKY75), had a large number of edges.

## Discussion

Gray mold caused by the necrotrophic fungus *B. cinerea* is one of the most devastating diseases in crop plants such as strawberry [33]. Infection development is a continuous process of host–pathogen interaction, and our understanding of how the plant invokes and transduces relevant signals from the local to the systemtic level and our attempts to design the best strategy for resistant cultivar development have been hampered by the heterogeneous nature of plant tissues [34]. The recent advent of single-cell transcriptomics in combination with laser-capture microscopy makes it possible to dissect the plant response at the level of individual cell types, identify cell-type-specific genes involved in signal transduction, and select promising candidate genes for gene editing and engineering. In pursuit of these goals, we have developed a single-cell transcriptome pipeline for studying the interaction between woodland strawberry (*F. vesca*) and *B. cinerea*.

### Construction of a single-cell atlas of strawberry leaves

*Fragaria vesca* ‘Hawaii4’, an ideal model plant, serves for cultivated strawberry and the Rosaceae family. In this study, we first captured the major cell types of woodland strawberry leaves to construct a high-resolution transcriptome atlas (Fig. 1b). Because there was no exact marker gene for each strawberry leaf cell type, we used multiple known orthologous marker genes from *Arabidopsis* [22, 29, 31, 35, 36] to annotate strawberry leaf cell types. Based on the conservation of gene expression patterns in strawberry and *Arabidopsis* and the verification of specific marker genes by laser microdissection, strawberry leaves could be separated into 12 cell types. Cells from the hydathode, upper epidermis, bundle sheath, xylem, phloem, lower epidermis, xylem parenchyma, and meristem could be clearly identified based on the marker genes of distinct cell clusters (Supplementary Table S4). Because there were not enough marker genes to determine its identity, we could not determine the cell type of cluster 7 (Fig. 1b). Interestingly, we detected the presence of meristem cells in the leaves, and we speculated that they may occur in vascular tissues [37].

### Distinct cell type responses to *B. cinerea* infection

Based on a single-cell expression atlas from three time points during *B. cinerea* infection, we showed that hydathode, upper epidermal, and mesophyll cells may mount the greatest initial response to infection. These results are similar to those of abiotic stress studies. When *Arabidopsis* roots experience heat stress, the hairs, non-hair epidermal cells, and cortex cells respond more quickly [16]. In rice seedlings, the mesophyll, parenchyma, and epidermal cells respond strongly to salt stress and nitrogen deficiency [38]. We observed that hydathode cells were enriched in the same pathways (e.g. response to biotic stimulus and defense response) at both 6 and 12 hpi, indicating that hydathode cells have already entered a defensive state at 6 hpi (Supplementary Fig. S6a). The upper epidermal cells and mesophyll cells actively respond to *B. cinerea* infection with distinct patterns of gene expression in each cell type. The normal function of mesophyll cells is primarily photosynthesis and light harvesting [27]; however, upon pathogen invasion their activities shifted to respond to infection, as suggested by enrichment in chitin catabolic process and defense response pathways (Figs 4e and 5c). The upper epidermal cells responded to pathogen invasion by activating the fatty acid biosynthetic process. Interestingly, cluster 3, including genes related to lipid biosynthesis process and xyloglucan metabolism, was expressed in the initial and later stages of the pseudotime trajectory (Supplementary Fig. S7c). The cuticle is an important part of the epidermis and may participate in the plant immune response, as shown in previous research [39]. Our results demonstrated that single-cell technology provides a high-resolution method for studies of heterogeneity in cell response and permits accurate detection of early pathogen responses.

### Gene expression patterns in *B. cinerea*-infected strawberry leaves

During plant–pathogen co-evolution, plants have evolved a complex immune defense system, including pattern-triggered immunity and effector-triggered immunity, function synergistically to optimize the plant systemic immune response [40, 41]. In this study, we found many genes related to recognition and signaling in the early stage of *B. cinerea* infection, such as LRR family protein and cysteine-rich receptor-like protein (Supplementary Table S9). Genes responding to stresses and involved in the secondary metabolism were identified upon *B. cinerea* infection, including peroxidase and pathogenesis-related protein (Supplementary Table S9). However, we found that not all cell types in the leaf tissue expressed the same disease resistance genes (Fig. 6, Supplementary Fig. S8). At 6 hpi, genes related to calmodulin, such as CML42 [42] were highly expressed in the hydathodes (Fig. 6a). By contrast, WRKY75 was highly expressed in upper epidermal cells (Fig. 7a, Supplementary Table S9), and has been shown to participate in jasmonate signaling pathways to regulate plant defense [43]. Interestingly, WRKY75 has also been classified as a specific marker gene in the root epidermis of *Arabidopsis*, where it participated in root system development [44]. ZAT11, a dual-function transcriptional regulator [45], was highly expressed in phloem (Fig. 7a, Supplementary Table S9). Previous studies have revealed that ZAT11 is involved in the formation of vascular tissue in a process mediated by the PXY transcriptional regulatory network [46]. These results implied that these differentially expressed genes not only played an important role in resistance to external pathogen stimuli to ensure plant growth, but functioned in plant growth and development. Even at 12 hpi, we also detected small but important differences in the expression of upregulated genes (Figs 6b and 7b); for instance, most of the DEGs were enriched in the mesophyll cells, suggesting that these cells may serve as the main site of plant defense responses.

To identify the role of TFs in plant disease response, we analyzed the expression of TFs in distinct cell types. HSP90.7, BIP2, JAZ2, ANAC002, WRKY75, and ZAT11 may act as key transcription factors to maintain communication between cell types at 6 hpi (Fig. 7b, Supplementary Table S9). WRKY, HSF, NAC, TIFY, ERF, bHLH, C2H2, and MYB family genes were identified as differentially expressed in cell types at 12 hpi (Fig. 7c, Supplementary Table S9). Co-expression network analysis suggested that these TFs do not act alone but may form a powerful immune network. Taken together, these results indicate that each cell type actively participates in the transcriptional regulation process through distinct expression patterns, and different cell types may communicate with each other to build a complex transcriptional regulatory network to resist *B. cinerea*.

In conclusion, we established markers related to different cell types of strawberry leaves and constructed a high-resolution single-cell gene expression atlas for the early process of strawberry response to *B. cinerea* infection. This is the first report of a single-cell transcriptome in woodland strawberry leaves, and the cell-type markers developed here can be used to separate heterogeneous tissues into more specific cell types, not limited to *Arabidopsis* and field crops [34, 47]. These findings lay a foundation for further investigation of the dynamic process of *B. cinerea* infection and the functional characterization and manipulation of candidate genes to develop resistant cultivars.

## Materials and methods

### Plant material, growth conditions, and fungal treatment

Woodland strawberry (*F. vesca* ‘Hawaii4’) was used for the scRNA-seq experiment. Seeds were sown on Murashige and Skoog medium containing 1.0% sucrose under a light intensity of 100 μmol m^−2^ s^−1^ and long-day conditions (16 h light/8 h dark) at 25°C. For pathogen infection, *B. cinerea* strain Bc05.10 was cultured on CM agar plates in the dark at 25°C as previously described [48]. Fifteen-day-old seedlings were sprayed with conidial suspensions containing 10^6^ spores/ml in SMB buffer (10 g/l mycological peptone, 40 g/l maltose). The first true leaf tissues were harvested at 0, 6, and 12 hpi for immediate protoplast isolation; each sample contained 50 pieces of true leaf tissue.

### Scanning electron microscopy

Leaf samples collected at 0, 6, and 12 hpi were fixed separately in 2.5% glutaraldehyde. After 8 h, the samples were washed three times with 0.1 M phosphate buffer, dehydrated in an ethanol gradient (50, 70, 80, and 90%) that was replaced three times with *tert*-butanol, and dried in a freeze-dryer. The samples were covered with a 10-nm gold film using an ion sputtering instrument (MC1000, Hitachi, Japan) and observed under a scanning electron microscope (SU8010, Hitachi, Japan).

### Protoplast isolation and scRNA-seq library construction

Leaf tissues were cut into pieces and placed in RNase-free enzyme solution (2% cellulose R10, 0.6% macerozyme R10, 0.8 M mannitol, 40 mM KCl, 20 mM CaCl_2_, 40 mM MES, 0.05% β-mercaptoethanol, and 0.1% bovine serum albumin). The tissues were enzymolyzed at 100 rpm for 4 h at 26°C in the dark, and the protoplasts were purified in W5 solution [0.08 M MES (pH 5.5), 0.1 M KCl, 0.02 M MgCl_2_, 0.4 M mannitol, and 0.1% bovine serum albumin], strained twice through a 38.5-μm filter, centrifuged at 200 g for 6 min, and washed twice with 8% mannitol to obtain pure protoplasts. The density of the protoplasts was determined with a hemocytometer and adjusted to 700–1200 cells/μl. The activity of single-cell suspensions was detected by fluorescein diacetate staining and trypan blue staining, and protoplasts with >90% activity were selected for future analysis.

The scRNA-seq libraries were processed on the 10x Chromium 3′ Single Cell platform (10x Genomics, Pleasanton, CA, USA). Briefly, using a microfluidic chip, single-cell suspension and beads containing a cell barcode were enclosed in droplets to form a single-cell GEM (gel beads in emulsion) structure. mRNA of the cell undergoes reverse transcription reaction in the droplet to form cDNA and construct the cDNA library. Single-cell FASTQ sequencing reads were mapped to the woodland strawberry ‘Hawaii 4’ reference genome (*F. vesca* v4.0.a1) [49]; the genome annotation has a total of 28 588 gene models. The read mapping data were converted to digital gene expression matrices using the Cell Ranger single-cell software suite (v3.1.0) provided by the 10x Genomics website.

### scRNA-seq data dimensionality normalization and clustering

The raw count matrices were analyzed using the Seurat package (v3.2.0) in R (v4.0.2). Before analyzing the scRNA-seq data, standard preprocessing steps were performed to remove dead and bimodal cells and to filter out cells with >0.05% mitochondrial and ribosomal sequences. The number of raw gene counts from each cell was normalized relative to the total number of counts, and we identified the top 2000 highly variable genes for use in cluster analysis. Principal component analysis (PCA) was used for dimensionality reduction. The RunTSNE function was used to visualize cell clusters, FindAllMarkers was used to identify cluster-enriched genes (marker genes), and FindIntegrateData was used to integrate the data from the three samples. CCA+ anchors (Seurat v3) were used to remove batch effects between different samples [50].

The FindMarkers function in Seurat was used to identify DEGs between samples based on a dual threshold of | log_2_FC | ≥ 0.8 and false discovery rate (FDR)  ≤ .01 [51]. The intersections of differentially expressed genes between samples were visualized using the UpSetR function in R [52].

### Comparison of interspecies scRNA-seq data

To identify different cell types, we used known marker genes from *Arabidopsis* to annotate strawberry cell types. Orthofinder was used to cluster single-copy orthologous protein sequences between woodland strawberry and *Arabidopsis*. The protein sequences were downloaded from the GDR (https://www.rosaceae.org) and TAIR (https://www.arabidopsis.org) websites

To integrate the single-cell data from *Arabidopsis* and woodland strawberry, we downloaded the published *Arabidopsis* leaf scRNA-seq data from the NCBI (GSE161482). We used sctransform to normalize and standardize the differences between the *Arabidopsis* and strawberry scRNA-seq data according to a published method [53]. After t-SNE dimensionality reduction, eight cell clusters were obtained. The AverageExpression function was then used to calculate the average gene expression level of each cluster. The cluster relationships between woodland strawberry and *Arabidopsis* were represented by Pearson’s correlation coefficients.

### Gene ontology term analysis

Marker genes and differentially expressed genes in specific samples were annotated using biological process of GO terms. GO terms and functional annotations were assigned based on the *Fragaria_vesca*_v4.0a1_go file of the *F. vesca* v4.0.a1 genomes. The clusterProfiler R package was then used to perform GO enrichment analyses with default parameters [54].

### Bulk RNA-seq

The first true leaves of 3-day-old woodland strawberry uninfected seedlings were harvested for RNA extraction. Total RNA was extracted from unprotoplasted and protoplasted leaf tissues using the Plant Total RNA Isolation Plus Kit (Foregene, Chengdu, China). Each sample contained three replicates. One microgram of RNA was used to construct mRNA libraries using the chain-specific method with the NEBNext^®^ Ultra™ Directional RNA Library Prep Kit (NEB, USA) following the manufacturer’s recommendations. The cDNA library was sequenced using the Illumina NovaSeq platform to generate 150-bp/150-bp paired-end reads. Raw reads in FASTQ format were firstly processed through Perl scripts. After preprocessing and quality control, the clean reads were mapped to the *F. vesca* v4.0.a1 genome using HISAT2 with default parameters. DEGs were identified using DESeq with a dual threshold of |log_2_FC| ≥1 and FDR ≤ .01. The log_2_ (mean RPM + 1) and Pearson correlation coefficients of bulk and single-cell RNA sequence data were calculated in R.

### Laser capture microdissection and RT–qPCR analysis

The first true leaves were embedded on a cryostat (Leica, Germany) using optimal cutting temperature compound, then immediately frozen. The embedded blocks were trimmed, sliced (18 μm thickness), transferred to a Leica PET membrane 1.4-μm microscopy slide, and dehydrated with 100% absolute ethanol. Laser microdissection was performed with the Leica Microsystems CMS system. The first true leaves of strawberry were divided into four cell types: upper epidermal, lower epidermal, mesophyll, and vascular tissue. Approximately 300 pieces of each cell type were pooled for immediate RNA extraction.

Total RNA was extracted from different cell types using the Arcturus PicoPure RNA Isolation Kit (Applied Biosystems, USA); there were three replicates of each cell type and 0, 6, and 12 hpi inoculation treatments. The relative expression of tissue-specific genes and differentially expressed genes in response to disease was measured by RT–qPCR; these genes had been identified in the scRNA-seq experiment. cDNA was synthesized using the PrimeScript RT Reagent Kit with gDNA Eraser (Takara, Dalian, China). The RT–qPCR primers are listed in Supplementary Table . RT–qPCR was performed using SYBR Premix Ex Taq II (TaKaRa) on a Light Cycler 480 II instrument (Roche). Relative gene expression values were calculated by the 2^−ΔΔCt^ method.

### Pseudotime analysis

The single-cell data were converted to a Cell Data Set object with the as.CellDataSet function in Seurat. Monocle 2 was used to reconstruct the pseudotime developmental trajectory in R [55]. The estimateSizeFactorsand and estimateDispersions functions were used to standardize the differences between cells. We used differentialGeneTest to select genes that define cell processes, the setOrderingFilter function to mark the ordered genes, and the DDRTree method to perform dimensionality reduction processing. The cells were sorted twice with the orderCells function and the root_state parameter was set for the second execution of the orderCells function. Cell pseudotime development trajectories were visualized using the plot_cell_trajectory function.

### Co-expression network of differentially expressed transcription factors

Differentially expressed transcription factors were analyzed at specific lesion time points (| log_2_FC | ≥ 0.8 and FDR ≤ 0.01). First, we connected highly expressed TFs of each cell type at 6 hpi in a protein–protein interaction network using the STRING database. Then, we calculated the Pearson correlation coefficients (PCCs) between differentially expressed transcription factors at 12 hpi and constructed a co-expression network using Cytoscape_v3.7.2 (PCCs ≥ 0.6, *P* < 0.05).

## Supplementary Material

Web_Material_uhab055

## Data Availability

All high-throughput sequencing data have been deposited in the Genome Sequence Archive in National Genomics Data Center, China National Center for Bioinformation, Chinese Academy of Sciences, under accession number CRA004848 (https://ngdc.cncb.ac.cn/gsa).
