# Supplementary material for: Development of a single-cell atlas for woodland strawberry (Fragaria vesca) leaves during early Botrytis cinerea infection using single-cell RNA-seq
Source: Hortic Res. 2022 Jan 19;9:uhab055. doi: 10.1093/hr/uhab055 (PMC8969069; doi:10.1093/hr/uhab055)
Supplement: Web_Material_uhab055 [file web_material_uhab055.zip › Supplemental Figure.docx]

**
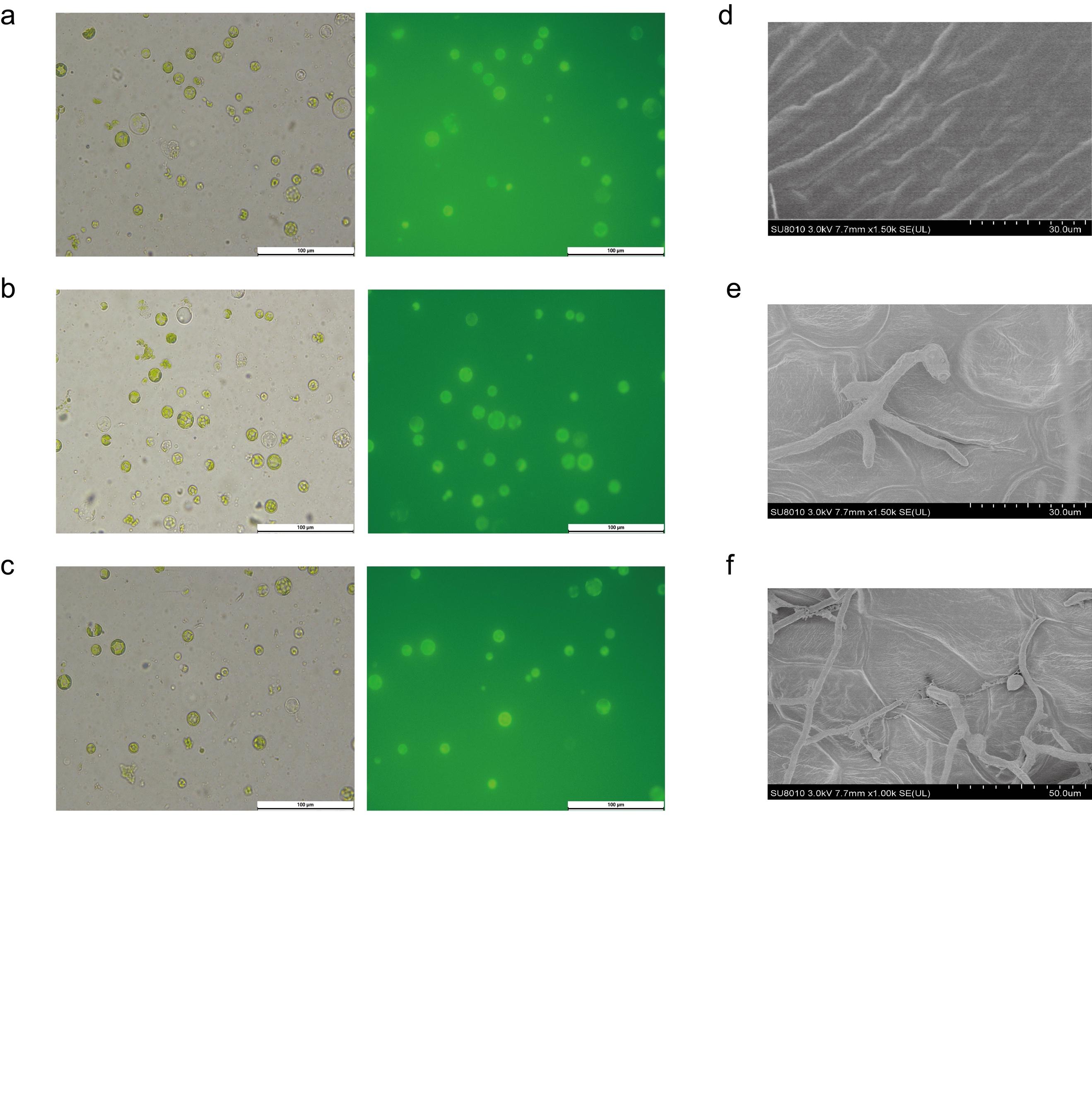
**

**Fig. S1.** **Strategies to enrich woodland strawberry leaf protoplasts.** **a** After separation of protoplasts from leaf samples in an enzyme solution (left), fluorescein diacetate staining was used to detect the activity of the protoplasts (right). **b** Protoplasts were isolated from leaf samples in an enzyme solution at 6 hpi (left), and fluorescein diacetate staining was used to detect the activity of the protoplasts (right). **c** Protoplasts were isolated from leaf samples in an enzyme solution at 12 hpi (left), and fluorescein diacetate staining was used to detect the activity of the protoplasts (right). **d–f** Scanning electron microscope analysis of leaf infection by *B. cinerea* in Mock (**d**), S6 (**e**), and S12 (**f**) samples.

**
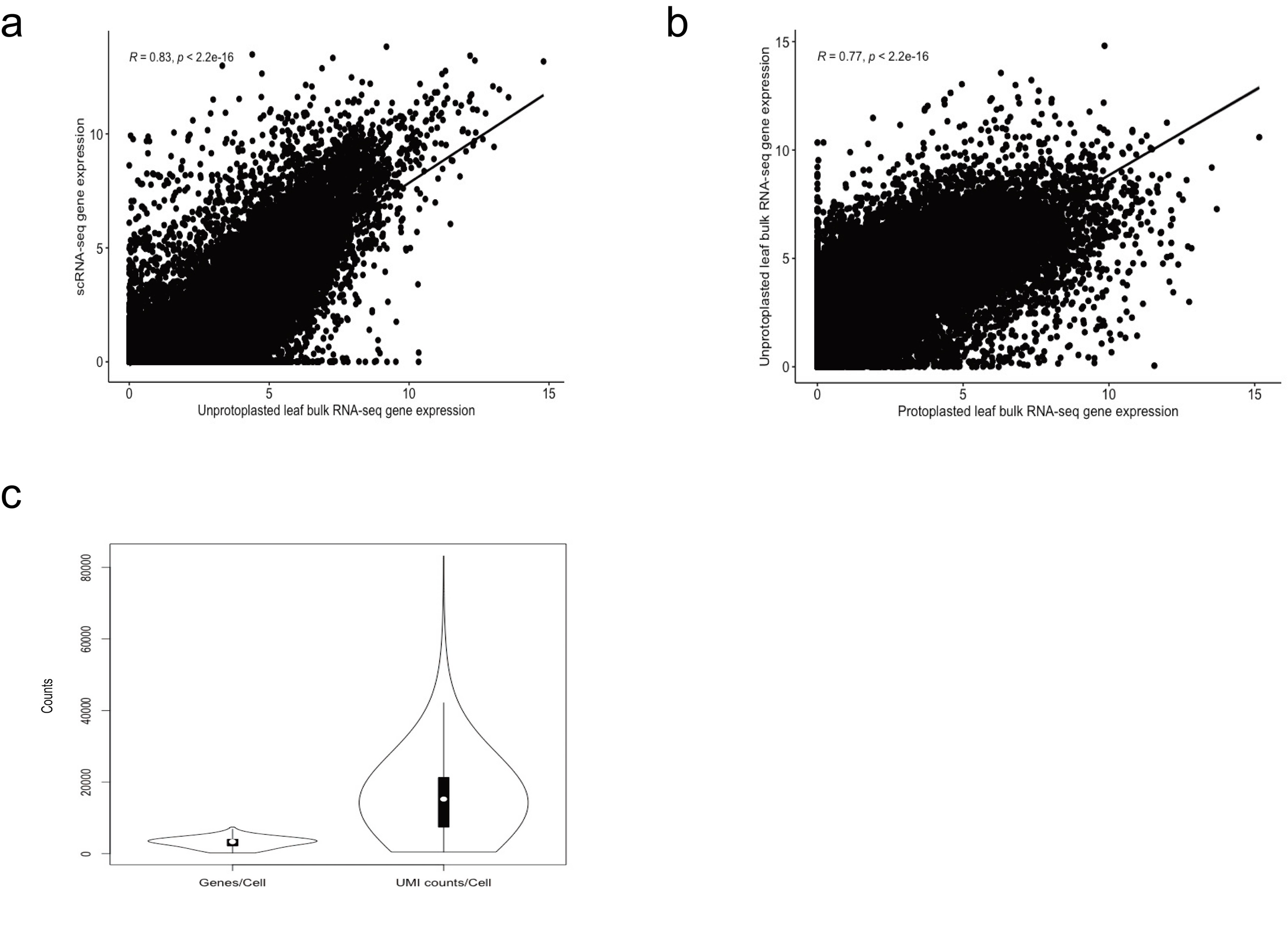
**

**Fig. S2. ScRNA-seq and bulk RNA-seq of woodland strawberry leaves.** **a** Correlation analysis of scRNA-seq and unprotoplasted leaf bulk RNA-seq gene expression**. b** Correlation analysis of protoplasted and unprotoplasted leaf bulk RNA-seq gene expression**. c** Violin plot comparing gene and UMI counts per cell.

**
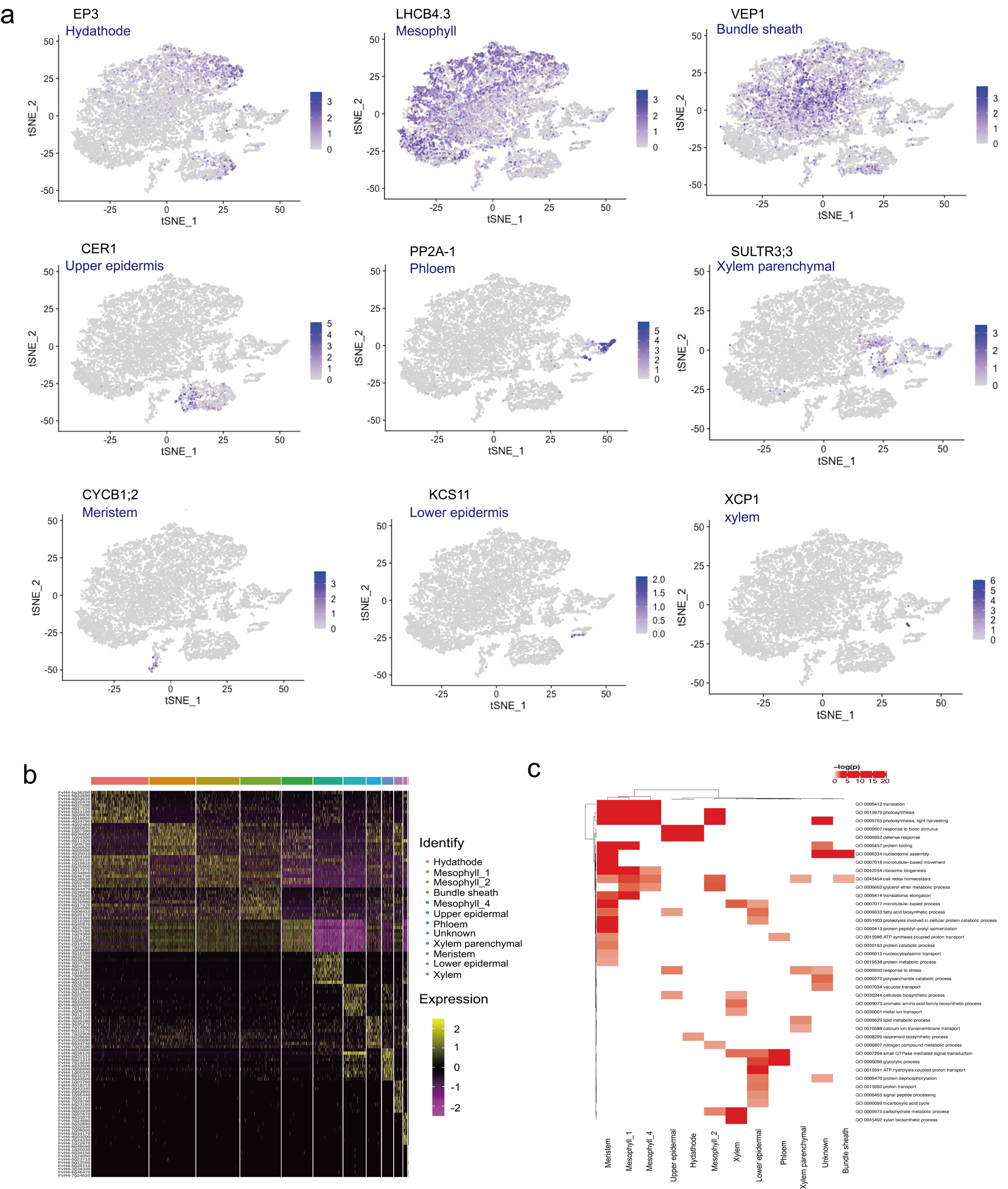
**

**Fig. S3. Analysis of cluster-specific marker gene expression in *F. vesca* leaves. a** t-SNE plot showing the expression of representative cluster-specific marker genes in woodland strawberry leaves. Color bars represent the gene expression level. **b** Heatmap showing the expression of the top 10 cluster-specific marker genes in woodland strawberry. Color bar represents the gene expression level. **c** GO enrichment heatmap for cluster-specific genes (FDR ≤ 0.05).


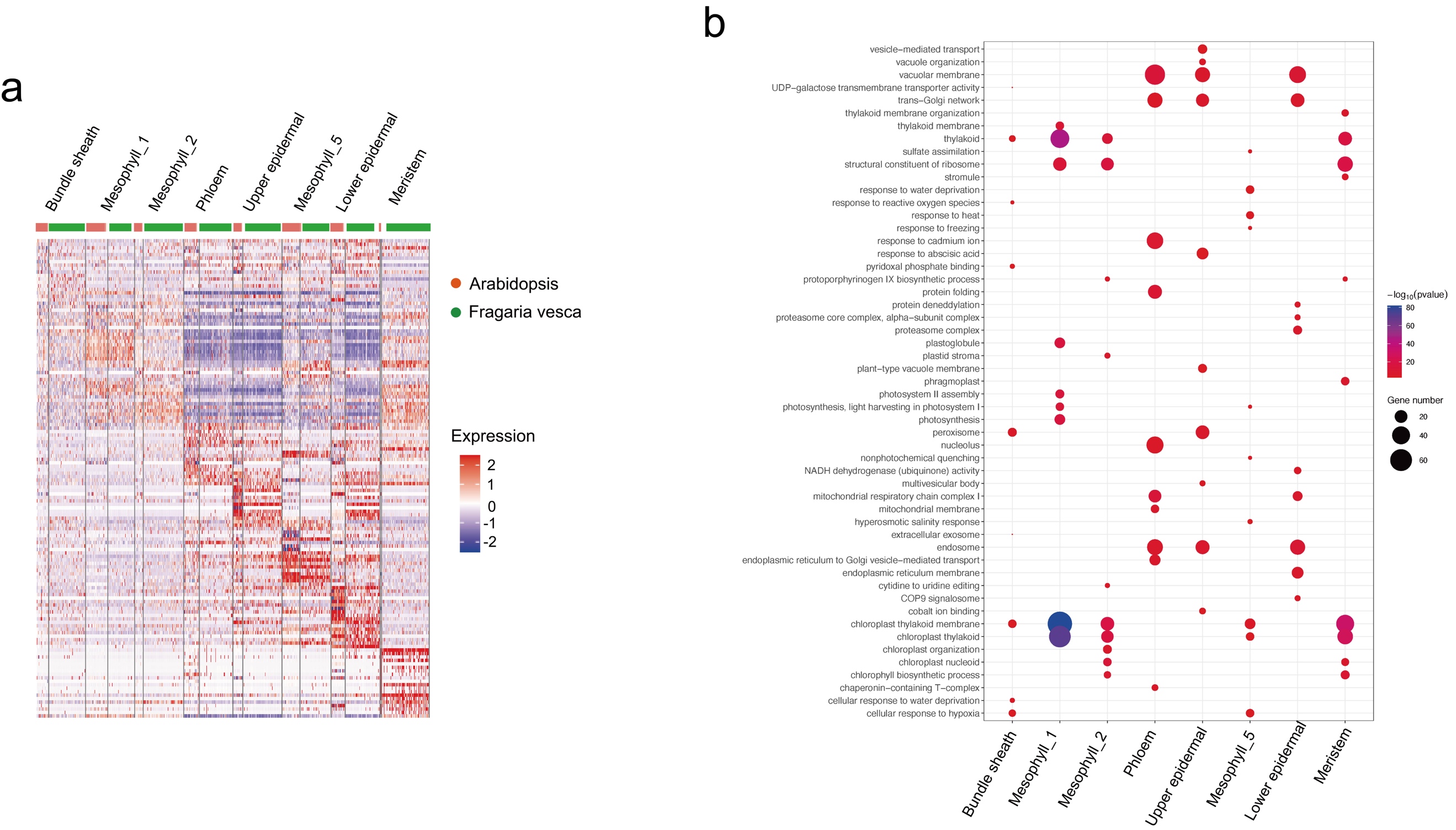


**Fig. S4. Conserved patterns of gene expression in cell types of woodland strawberry and *Arabidopsis*.** **a** Heatmap showing similar expression patterns of orthologous marker genes for each cell type in *F. vesca* and *Arabidopsis*. **b** GO enrichment analysis of marker genes for each cell type.

**
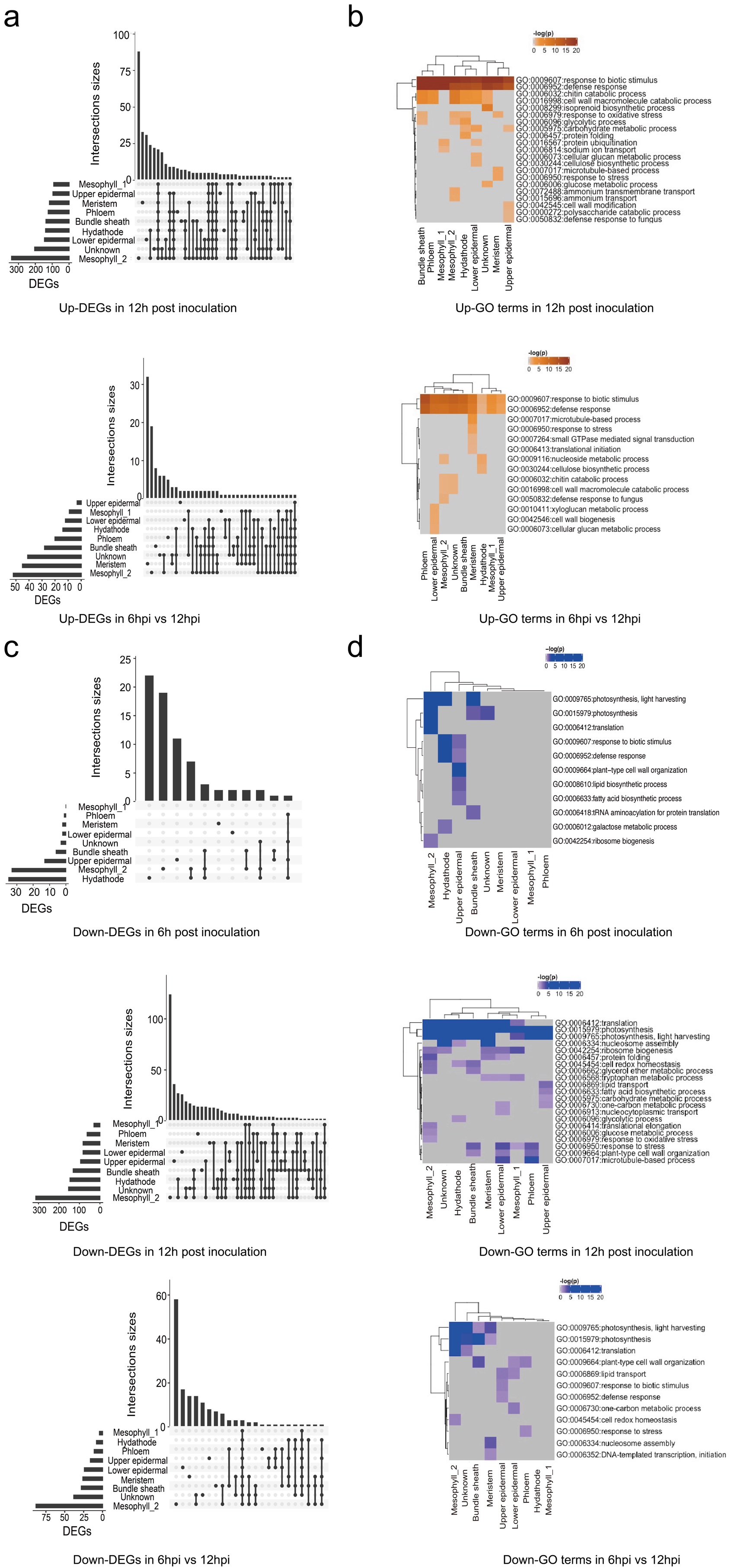
**

**Fig. S5. DEGs and enriched GO terms in response to *B. cinerea* infection in individual cell types. Similar to Fig. 3e, f. a** Bar plots of upregulated DEGs in Mock vs S12 (top) and S6 vs S12 (bottom). **b** GO enrichment heatmap of upregulated DEGs in each cell type for Mock vs S12 (top) and S6 vs S12 (bottom). **c** Bar plots of downregulated DEGs in Mock vs S6 (top), Mock vs S12 (center), and S6 vs S12 (bottom). **d** GO enrichment heatmap of downregulated DEGs in each cell type for Mock vs S6 (top), Mock vs S12 (center), and S6 vs S12 (bottom).

**
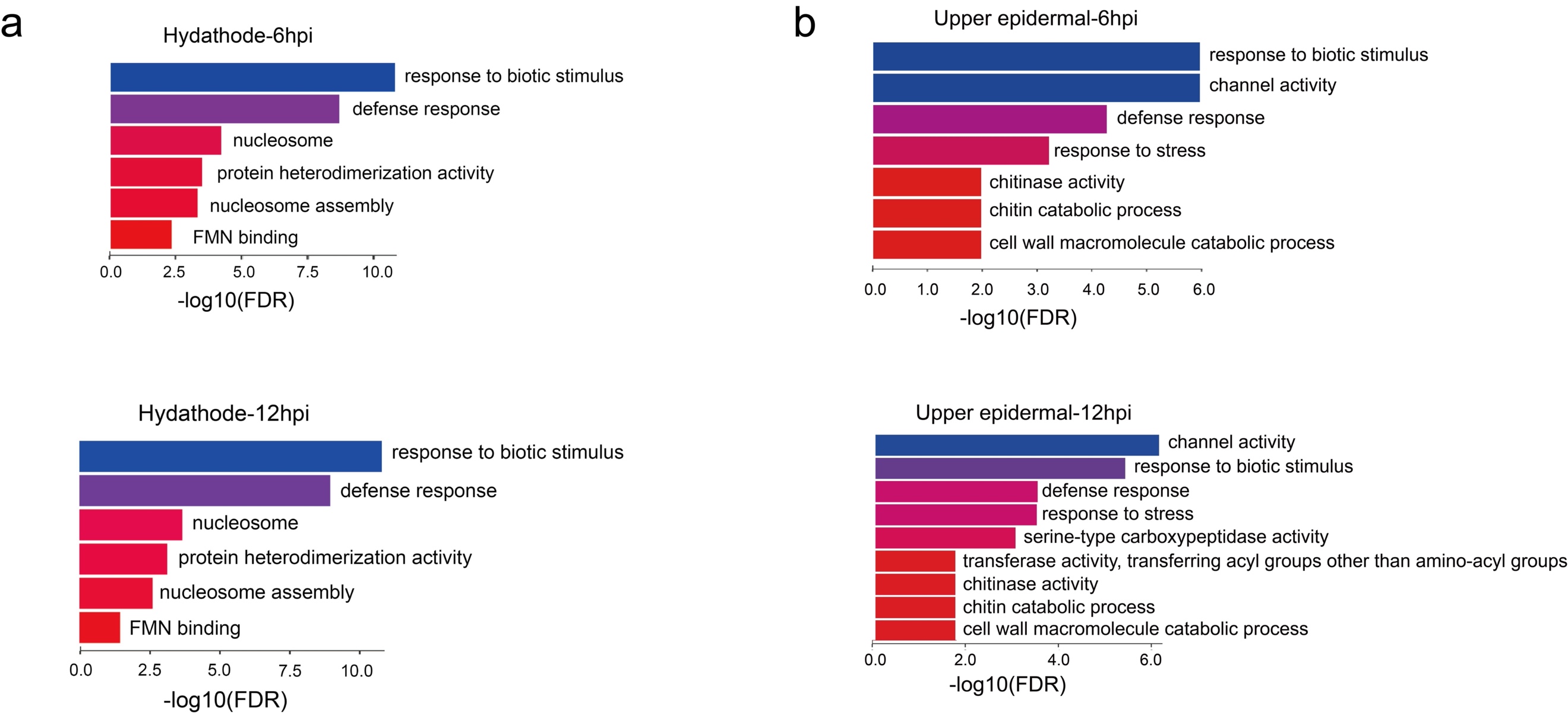
**

**Fig. S6. Enriched GO terms in hydathode and upper epidermal cells at 6 and 12 hpi. a** The top enriched pathways in hydathode cells at 6 hpi (top) and 12 hpi (bottom). **b** The top enriched pathways in upper epidermal cells at 6 hpi (top) and 12 hpi (bottom).

**
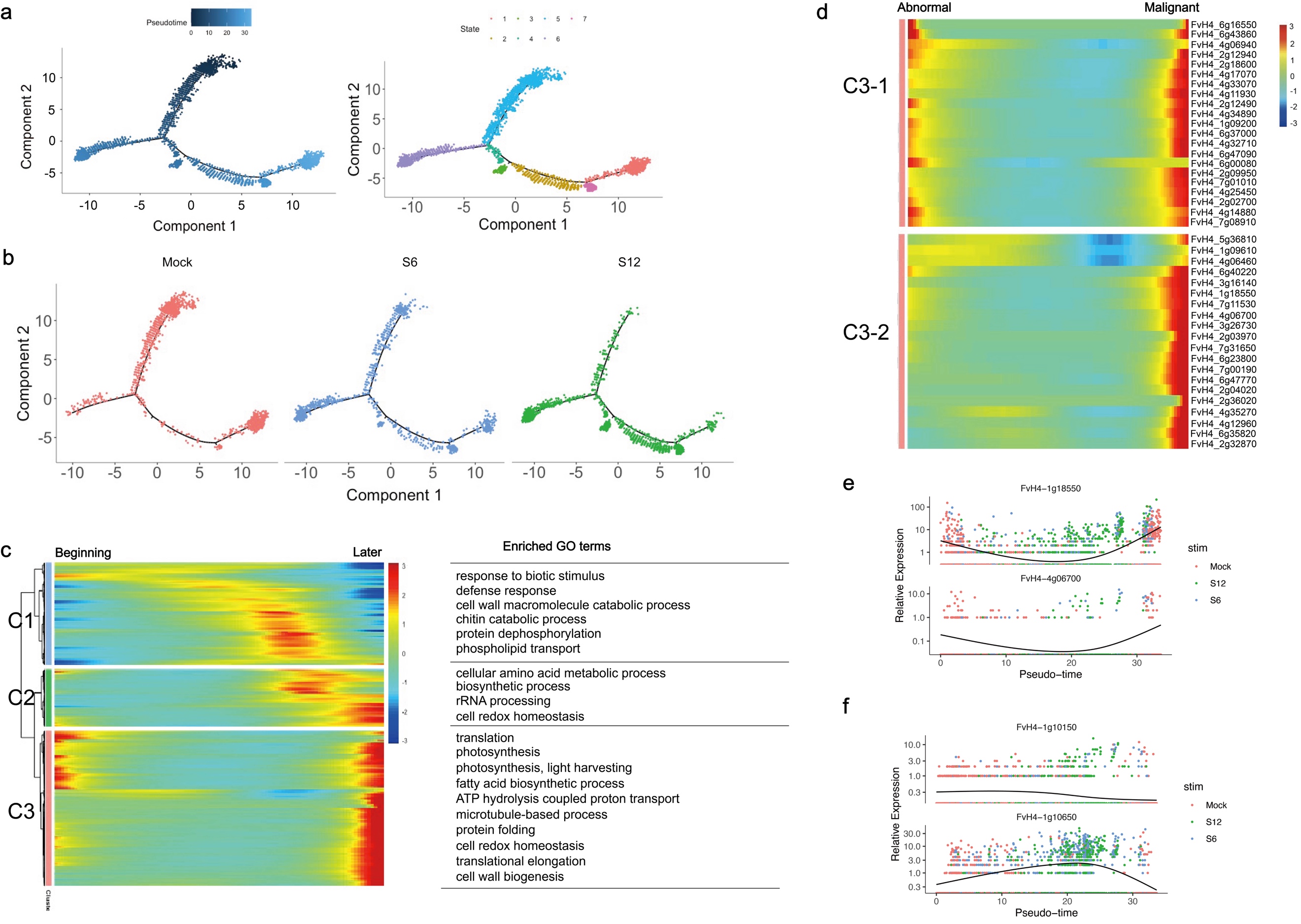
**

**Fig. S7.** **Pseudotime analysis of epidermal cells from different samples. Similar to Fig. 5. a** Developmental trajectory of epidermal cells. Each dot represents a single cell. The color of left picture represents the pseudotime score. The colors of right picture represent different states. **b** The upper epidermal cells distribution on the trajectory for Mock, S6, and S12. **c** Pseudotime heatmap of GO analysis for differentially expressed genes (FDR ≤ 0.05). The differentially expressed genes (rows) along the pseudotime (columns) were hierarchically clustered into three profiles. Representative gene functions and pathways for each profile were shown. Color bar indicates the relative gene expression level. **d** Heatmap showing the expression of representative identified genes from the third profile, clustered into two subgroups. Color bar indicates the relative gene expression level. **e–f** Gene expression kinetics along a pseudotime progression for representative genes in the beginning **(e)** and later **(f)** infection stage.

**
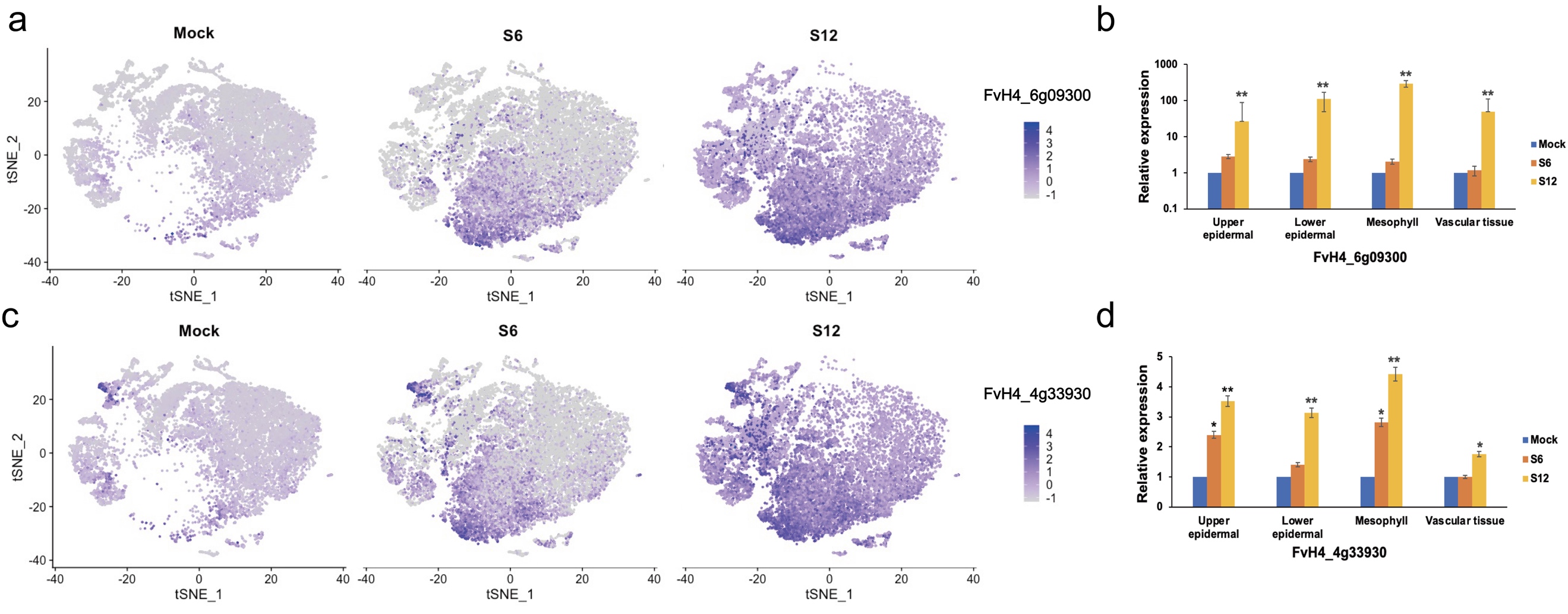
**

**Fig. S8. Distribution and expression of disease-related genes. Similar to Fig. 6. a, b** FeaturePlot **(a)** and RT-qPCR **(b)** showing the expression distribution of *FvH4_6g09300* (CML42) in different processing stages (Student’s *t-*test, **P*<0.05 and ***P*<0.01). **c, d** FeaturePlot **(c)** and RT-qPCR **(d)** showing the expression distribution of *FvH4_4g33930* (PCR2) in different processing stages (Student’s *t-*test, **P*<0.05 and ***P*<0.01).
