# Supplementary material for: Development of a single-cell atlas for woodland strawberry (Fragaria vesca) leaves during early Botrytis cinerea infection using single-cell RNA-seq
Source: Hortic Res. 2022 Jan 19;9:uhab055. doi: 10.1093/hr/uhab055 (PMC8969069; doi:10.1093/hr/uhab055)
Supplement: Web_Material_uhab055 [file web_material_uhab055.zip › Supplemental Table legends.docx]

**Table S1.** scRNA-seq data and bulk RNA data generated in this study and differentially expressed genes (DEGs) in protoplasted and unprotoplasted leaf samples.

**Table S2.** List of cluster-enriched genes for mock sample.

**Table S3.** Known marker genes used for cluster annotation.

**Table S4.** List of single copy orthologue between woodland strawberry and Arabidopsis.

**Table S5.** List of enriched genes for each cluster of integrate scRNA-seq data.

**Table S6.** List of differentially expressed genes between each sample for each cluster.

**Table S7.** Enriched pathways for differentially expressed genes between hydathode, mesophyll_1 and upper epidermal.

**Table S8.** Primers used in this study.

**Table S9.** Defense-related genes (corresponds to Fig. 6) and transcription factors (corresponds to Fig. 7) during lesion development.
